# Focused Assessment With Sonography in Trauma (FAST) as a Prognostic Tool in Blunt Abdominal Trauma: A Prospective Observational Study

**DOI:** 10.7759/cureus.93071

**Published:** 2025-09-23

**Authors:** Daniel E, Srinivasan C, Karthikeyan Selvaraj

**Affiliations:** 1 Department of General Surgery, Sree Balaji Medical College and Hospital, Chennai, IND

**Keywords:** abdominal blunt trauma, fast (focused assessment with sonography for trauma), mortality, triage, ultrasound imaging

## Abstract

Introduction

Blunt abdominal trauma (BAT) is a significant contributor to trauma-related morbidity and mortality globally. While Focused Assessment with Sonography for Trauma (FAST) is widely utilized for early diagnosis, its role in predicting patient outcomes requires further evaluation.

Methods

A prospective observational study involving 80 BAT patients was conducted at a tertiary trauma center over 18 months. All patients underwent FAST at admission. Correlations between FAST results and clinical outcomes such as surgical intervention, ICU stay, morbidity, mortality, and hospital stay duration were assessed. CT was used as the diagnostic gold standard. Statistical analyses included sensitivity, specificity, logistic regression, and comparison of outcome variables.

Results

Out of 80 patients, 36 (45%) had a positive FAST. FAST showed a sensitivity of 79.1%, specificity of 94.6%, positive predictive value (PPV) of 94.4%, and negative predictive value (NPV) of 79.5% compared to CT. Surgical intervention (72.2% vs. 9.1%), ICU admission (50% vs. 11.3%), morbidity (38.9% vs. 6.8%), and mortality (13.9% vs. 2.3%) were significantly higher in FAST-positive patients (p<0.001). Hospital stay was significantly longer in FAST-positive patients (10.2 ± 3.8 vs. 6.1 ± 2.9 days, p<0.001).

Conclusion

FAST is an effective prognostic tool in BAT. A positive FAST result strongly correlates with adverse outcomes and supports early triage, surgical planning, and ICU utilization.

## Introduction

Blunt abdominal trauma (BAT) is a significant cause of trauma-related morbidity and mortality, accounting for up to 20% of major trauma admissions globally [1]. Motor vehicle collisions, falls, and assaults are common causes, often leading to injuries of the liver, spleen, kidneys, and hollow viscera of the abdomen [2]. In many cases, internal bleeding occurs without external signs, complicating early diagnosis and delaying treatment [3].

While computed tomography (CT) remains the gold standard for identifying intra-abdominal injuries, it is not used in unstable patients or low-resource settings due to cost, accessibility, and the need for patient transport [4]. Focused Assessment with Sonography for Trauma (FAST) has emerged as a non-invasive, bedside modality capable of detecting free intraperitoneal fluid rapidly. It is incorporated in the Advanced Trauma Life Support (ATLS) guidelines and has become a standard part of trauma evaluations [5]. FAST evaluates four anatomical regions: hepatorenal space (Morison’s pouch), splenorenal recess, pelvis (Douglas pouch in women and rectovesical pouch in men), and pericardium. It has been shown to have high specificity in detecting hemoperitoneum, but variable sensitivity, often dependent on operator skill and patient condition [6]. Nevertheless, the prognostic value of FAST remains underexplored.

Recent literature suggests that positive FAST findings are associated with worse clinical outcomes, such as increased need for surgical intervention, ICU care, and higher mortality [7]. Dammers et al. reported that FAST was a stronger predictor of adverse outcomes than injury severity scores in stable trauma patients [8]. Similarly, Rowell et al. noted that a negative FAST does not reliably exclude intra-abdominal injury, particularly in hypotensive patients [9]. Given the high stakes of trauma triage, particularly in low-resource or high-volume settings, the prognostic application of FAST could enhance clinical decision-making. This study assesses the prognostic value of FAST in patients with BAT by correlating initial scan results with outcomes, including surgery, ICU admission, hospital stay, and mortality. FAST has demonstrated utility in hemodynamically stable patients with BAT. In addition, ultrasound has been shown to be more cost-effective when compared with diagnostic peritoneal lavage (DPL) or CT scanning [10].

The primary aim of this study is to investigate the role of the FAST as a predictor of clinical outcomes in patients with blunt abdominal trauma, with these key objectives in mind: (i) To assess the diagnostic accuracy of FAST in detecting various abdominal injuries, (ii) To evaluate the impact of FAST results on clinical decision-making, including the choice of treatment modalities, and (iii) To examine the association between FAST findings and patient outcomes, including morbidity, mortality, length of hospital stay, and healthcare resource utilization.

## Materials and methods

This was a prospective, observational study conducted at the Department of General Surgery, Sree Balaji Medical College and Hospital, Chennai, Tamil Nadu, India. This study analysed FAST as a predictive tool in patients who presented with BAT. The observational design enabled real-time data collection and minimized recall bias while allowing accurate documentation of clinical outcomes associated with FAST findings.

The study was approved by the Sree Balaji Medical College and Hospital's Institutional Human Ethics Committee (approval number: 002/SBMCH/IHEC/2023/2080) and followed the guidelines laid by the Declaration of Helsinki regarding human subjects. All participants or their authorized representatives provided informed consent. Patient confidentiality was preserved through anonymization of data and secure data storage. No invasive interventions were performed specifically for the study. At any time, participants could discontinue their medical treatment and remain a part of the research.

Eligibility criteria

Patients diagnosed with BAT who had the FAST assessment as part of their first evaluation and came to the Trauma Care Unit were eligible for the study. 

Inclusion Criteria 

People of any gender or age who showed up with severe abdominal pain, not related to previous conditions, individuals who were evaluated for trauma initially, and who underwent FAST, and those who (or their legal guardians) provided informed consent for participation were included in the study.

Exclusion Criteria

Individuals with perforating abdominal injuries, patients who did not undergo FAST or had FAST performed after other diagnostic imaging, and patients (or legal guardians) who declined to participate or withdrew consent were excluded from the study.

Sampling technique and sample size

A purposive sampling method was used. This non-probability sampling approach was appropriate given the specific clinical context and targeted patient group.

Sample size was calculated with the following formula: $n = \frac{4pq}{d^{2}}$, where p = prevalence of positive FAST findings in BAT (14.7%), q = 1-p = 85.3, d = absolute error (8%), Z = 1.96 for 95% confidence interval (CI).

The resulting minimum sample size was 80 patients, which was achieved during the study period.

Data collection

Data collection took place in the Trauma Unit and Emergency Department over a period of 18 months. A clinical proforma was used for data collection. The information gathered was categorized into the following domains: Demographic and clinical data, FAST Examination, Management, and Clinical outcomes.

Outcomes

Primary Outcome

The primary outcomes analysed were the correlation between FAST findings and key clinical outcomes: mortality, morbidity, and requirement for surgical intervention.

Secondary Outcomes

The secondary outcomes analysed included diagnostic accuracy of FAST compared to CT imaging, association between FAST findings and hospital resource utilization, including ICU stay and length of hospital admission, impact of FAST on routes for clinical decision-making, such as whether further imaging is necessary or whether treatment has to be adjusted. 

Statistical analysis

IBM SPSS Statistics for Windows, version 25 (IBM Corp., Armonk, New York, United States) was used to input and analyze data. A combination of descriptive and inferential statistics was used in the study. Mean ± standard deviation (SD) or median with interquartile range (IQR) were used to present continuous data, such as age and length of hospital stay. Categories such as gender and FAST results were represented using percentages and frequencies.* *

The relationships between FAST results and categorical outcomes, such as the necessity for surgery or ICU hospitalization, were examined using a chi-square test. In order to compare continuous variables between the FAST-positive and FAST-negative groups, the Mann-Whitney U test was used. The independent determinants of mortality and surgical intervention were identified using binary logistic regression. After controlling for any confounding variables, multivariate linear regression was used to determine the association between FAST results and continuous outcomes such as the length of time a patient spent in the hospital. For all comparisons, a p-value less than 0.05 was deemed statistically significant.

## Results

The study is done by comparing the patients who underwent FAST compared to those who were not, or had other radiological investigations done. 

Demographics and injury patterns

Among the 80 patients, 56 were male (70%) and 24 were female (30%). The majority were in the age group of 18-45 years (n=46), accounting for 57.5% of the total sample. The most common mechanism of injury was road traffic accidents (65%), followed by falls from height (17.5%) and assaults (11.3%), as shown in Table 1. 

**Table 1:** Distribution of participants by age groups and mechanism of injury (N=80)

Parameters	Frequency (Percentage)
Age Group (years)	
<18	7 (8.8%)
18–30	25 (31.3%)
31–45	21 (26.3%)
46–60	15 (18.8%)
>60	12 (15.0%)
Mechanism of injury	
Road Traffic Accident (RTA)	52 (65.0%)
Fall from Height	14 (17.5%)
Assault	9 (11.3%)

FAST diagnostic accuracy

FAST was positive in 36 patients (45%), as given in Table 2. When compared with Axial CT, which identified 43 patients with intra-abdominal injuries, FAST achieved a sensitivity of 79.1% and a specificity of 94.6%. The positive predictive value (PPV) was 94.4% and the negative predictive value (NPV) was 79.5%.

**Table 2:** Correlation of FAST findings with CT scan results FAST: Focused Assessment with Sonography in Trauma

FAST Result	CT Positive	CT Negative	Total
Positive	34	2	36
Negative	9	35	44

These results show that FAST is highly specific and has strong predictive value in diagnosing free fluid but may miss subtle injuries, especially in the absence of significant hemoperitoneum.

Clinical outcomes based on FAST

Patients with a positive FAST had significantly worse clinical outcomes: Surgical intervention was required in 72.2% of FAST-positive patients versus only 9.1% of FAST-negative cases (p < 0.001). ICU admission was needed in 50% of FAST-positive patients compared to 11.3% in the negative group (p < 0.001). Morbidity was observed in 38.9% of FAST-positive patients vs. 6.8% among FAST-negative patients (p < 0.001). Mortality was significantly higher in FAST-positive patients (13.9%) than in those with a negative scan (2.3%; p = 0.035). Length of hospital stay was also greater: 10.2 ± 3.8 days in FAST-positive vs. 6.1 ± 2.9 days in FAST-negative patients (p < 0.001), as shown in Table 3. All these results were taken based on individuals who had mortality or morbidity associated with abdominal bleeding/injury. Mortality due to other factors of the patient's trauma, like head injury, chest injury, and blood loss outside the abdomen, was not taken into consideration.

**Table 3:** Comparison of clinical outcomes between FAST-positive and FAST-negative patients FAST: Focused Assessment with Sonography in Trauma; ICU: intensive care unit

Outcome	FAST-positive patients (n=36)	FAST-negative patients (n=44)	p-value
Surgery Required	26 (72.2%)	4 (9.1%)	<0.001
ICU Admission	18 (50.0%)	5 (11.3%)	<0.001
Morbidity	14 (38.9%)	3 (6.8%)	<0.001
Mortality	5 (13.9%)	1 (2.3%)	0.035
Hospital Stay (days)	10.2 ± 3.8	6.1 ± 2.9	<0.001

Injury patterns on CT

The liver and spleen were the most commonly injured organs, with multiple organ injuries occurring in 7.5% of patients. No significant injury was found in 38.8% of the cases, indicating that many patients with negative FAST and stable vitals could be managed conservatively, as shown in Table 4.

**Table 4:** Distribution of intra-abdominal organ injuries detected by CT in blunt abdominal trauma patients

Organ Injured	Frequency	Percentage
Liver	15	18.8
Spleen	13	16.3
Kidney	7	8.8
Bowel/Mesenteric	5	6.3
Bladder	2	2.5
Pancreas	1	1.3
Multiple Organs	6	7.5
No Significant Injury	31	38.8

Diagnostic performance analysis using a receiver-operating characteristic (ROC) curve

**Figure 1:**
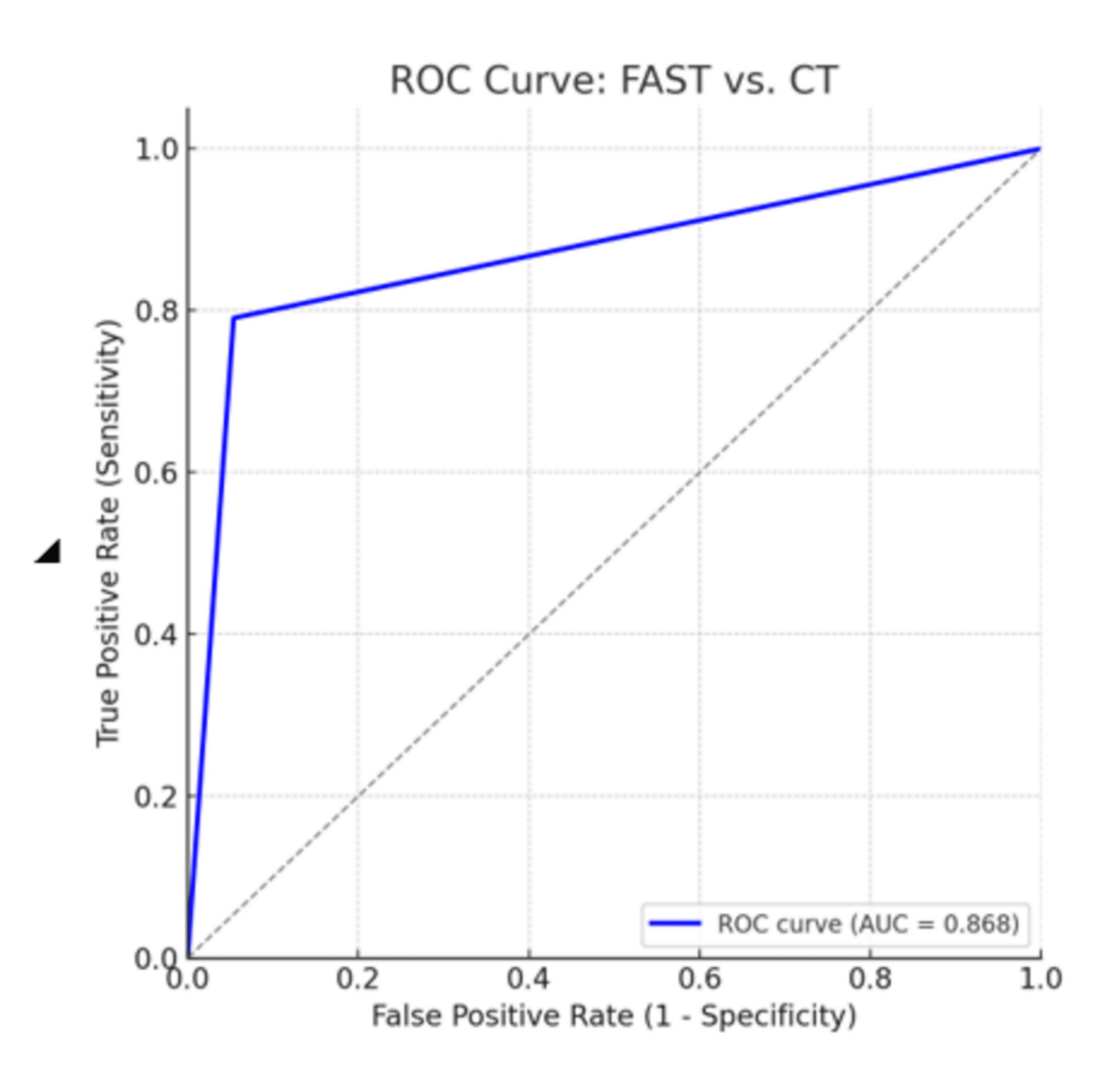
ROC Curve FAST: Focused Assessment with Sonography in Trauma; ROC: receiver operating characteristic

To further evaluate the discriminative ability of FAST as a diagnostic tool, a ROC curve was generated using CT scan findings as the gold standard. The ROC analysis revealed an area under the curve (AUC) of 0.917, indicating excellent diagnostic performance as shown in Figure 1.

This ROC curve demonstrates that FAST is highly accurate in identifying patients with intra-abdominal injuries. The high AUC value confirms its utility not only as a diagnostic modality but also as a triaging tool in the emergency setting.

## Discussion

This study underscores the prognostic utility of FAST in managing blunt abdominal trauma. Our findings show that a positive FAST result strongly correlates with adverse clinical outcomes, including surgical need, ICU admission, complications, and death. The results align with Dammers et al., who found that FAST had better predictive power for morbidity than injury severity scores [8]. Rowell et al. similarly noted that FAST could guide surgical decision-making in hypotensive patients but cautioned against ruling out injury solely on the basis of a negative FAST [9].

In our study, nine patients with negative FAST had CT-confirmed injuries, highlighting the limitations of relying on FAST alone. Our study's specificity and PPV (both over 94%) affirm FAST’s diagnostic value, as reported by Montoya et al. [4] and Zanobetti et al. [6]. However, its moderate sensitivity reiterates the importance of combining FAST with clinical judgment and follow-up imaging.

From a clinical standpoint, FAST offers major advantages in triage, especially in resource-limited environments. It helps prioritize patients for CT, surgery, or observation, reducing unnecessary delays and optimizing resource allocation.

Limitations of this study include its single-center nature and operator dependency in performing FAST. However, the consistent training and use of a standardized protocol likely minimized inter-observer variability. Overall, FAST has a sensitivity between 73% and 88% and a specificity between 98% and 100%, and is 96-98% accurate [10].

## Conclusions

FAST is a rapid, non-invasive, and highly specific tool that offers valuable prognostic information in BAT. A positive FAST is associated with significantly worse outcomes, including the need for surgery, ICU admission, and higher mortality. And this level of accuracy is independent of the practitioner performing the study. Surgeons, emergency physicians, ultrasound technicians, and radiologists all have similar results. Integrating FAST into trauma workflows can guide resource allocation and improve patient outcomes. Although FAST has these benefits, due to small sample size and specific criteria of the patients involved, the limitations of the generalizability of this study is acknowledged. 
